# Theoretical Modeling of the Redox Thermodynamics of Nucleic Acid Building Blocks in the Condensed Phase

**DOI:** 10.1021/acs.jpcb.5c03817

**Published:** 2025-08-29

**Authors:** Alessio Olivieri, Alessandro Nicola Nardi, Marco D’Abramo

**Affiliations:** † Department of Chemistry, Sapienza University of Rome, 00185 Rome, Italy; ‡ 27045 Nantes Université, CNRS, CEISAM UMR 6230, F-44000 Nantes, France

## Abstract

Low-energy solvated electrons can be captured by nitrogenous bases and localized on the nucleic acid to form a stable anion. The interaction between electrons and the bases is related to alterations in the stability and function of nucleic acids. Here, we report the theoretical-computational estimates of the adiabatic electron affinities (AEAs) and the reduction potentials of the nucleobases in solution. Our data show that pyrimidine bases exhibit the highest tendency to form stable anions in both the gas phase and condensed phases (water and *N*,*N*-dimethylformamide solutions). The addition of a ribose moiety increases the electron affinities of the nucleobases in the corresponding nucleosides across both environments. Finally, the comparison with the available experimental data shows that our QM/MM approach, based on a statistical-mechanical treatment of the system, is capable of furnishing accurate reduction-free energy differences in the condensed phase.

## Graphical abstract

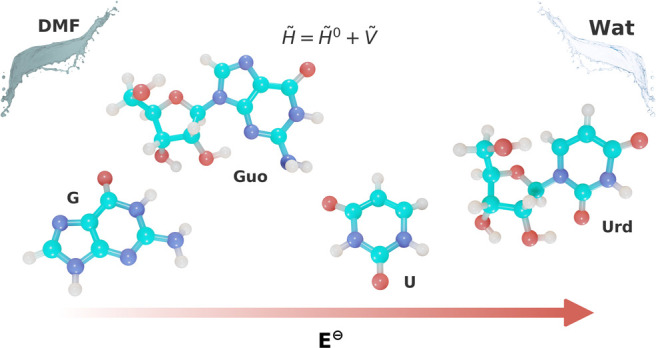

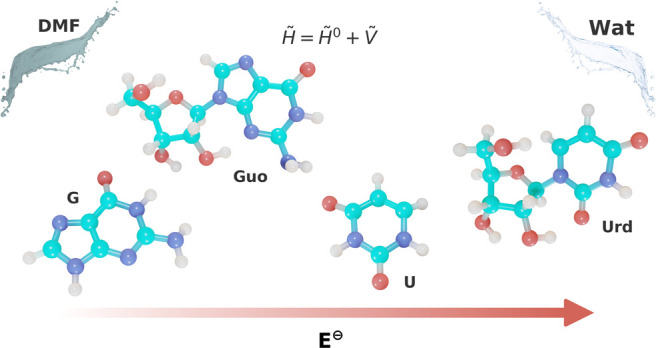

## Introduction

The interaction between electrons and the nucleobases of DNA and/or RNA is related to dissociative electron attachment, base modifications, strand breaks, and the formation of reactive intermediates.^1–5^ The generation of low-energy electrons results from the interaction between high-energy particles and the cellular matrix.^6–9^ These electrons can be captured by nucleic acids, resulting in alterations in their stability and function. Concurrently, charge migration along the double helix appears to be associated with genome repair mechanisms.^1–4,10^ Consequently, the interaction between electrons and nucleobases has been a subject of sustained interest since the late 1990s. Despite the concerted efforts of the scientific community, this process remains only partially understood.^11–21^ The paucity of available data on reductive damage to nucleic acid bases (NABs) is mainly attributable to the experimental and computational challenges associated with the study of the radical anionic species involved. The primary experimental challenge pertains to the presence of close-energy tautomers of the reduced bases. Furthermore, it is notable that a single tautomer may exhibit different charge-carrier behaviors, underscoring the complexity of the system.^3,12,15,22^ Additionally, the electron attachment to nucleobases can occur through two distinct mechanisms, leading to either a valence-bound (VB) or a dipole-bound (DB) state. The VB state has been conventionally described as the addition of an extra electron to the π* antibonding molecular orbital.^17,19^ Sufficiently polar molecules (in this context with a dipole moment equal to or greater than 2.5 D^23^) are capable of forming DB states, where an additional electron is loosely attached to the molecular framework through electrostatic charge-dipole interactions, and occupying a very diffuse orbital that extends outside the molecular region. Notably, the DB states almost maintain the molecular structure of their neutral precursors. The proximity of these states poses a significant challenge in the experimental characterization of the interaction between electrons and nucleobases, consequently leading to the availability of only a limited number of reliable estimates of electron affinities (EAs).^12,15,24,25^ From a theoretical and computational perspective, difficulties arise from the description of the correct nature of the radical anion state and the free electrons involved in the process. Accurate computation of EAs for such species requires quantum chemical methods employing high-quality basis sets augmented with diffuse functions.^17,18,26–29^ Nevertheless, a number of experimental and computational endeavors have been undertaken in this regard. In several experimental works, a variety of techniques have been employed, including electron transmission spectroscopy (ETS),^12^ Rydberg electron transfer (RET) spectroscopy,^14,23,24^ photodetachment-photoelectron spectroscopy (PD–PES),^16,25,30–32^ and low-energy photoelectron transmission (LEPET) spectroscopy,^33,34^ to investigate the EA of the nucleic acid building blocks. The vertical electron affinity (VEA) of adenine, cytosine, thymine, and uracil, in the gas phase, as determined by ETS and RET spectroscopy, ranges from −0.22 to −0.55 eV.^12,15,22^ The values of the experimental adiabatic electron affinities (AEAs) of cytosine, thymine, and uracil are slightly positive (ca. 0.10 eV).^13^ In contrast, the AEAs of adenine and guanine were found to be negative.^16,31^ Additionally, the reduction potentials of nucleobases in *N*,*N*-dimethylformamide (DMF) have been reported by Seidel et al.,^11^ providing valuable insights into the redox behavior of these bases. In parallel with experimental studies, a variety of computational methods have been employed over the years to address the problem of modeling the electron attachment to NABs.^3,17,26,28,29,35–38^ The 1970s witnessed the inception of early quantum chemical studies, characterized by systematic efforts to describe the gas phase reduction of DNA/RNA subunits by Hartree–Fock (HF) methods.^39–41^ However, early works showed that high-level correlated ab initio methods (i.e., CCSD­(T) and CASPT2) can provide reliable results.^17,28,29^ The combination of these sophisticated theoretical methods with extended basis sets—necessary to accurately treat radical anion species—gives rise to computationally demanding calculations. Notably, recent advances in density functional theory (DFT) have demonstrated the capability of selected density functionals (e.g., M06-2X) to reproduce experimental electron affinity values with comparable accuracy with respect to high-level correlated methods.^18^ For example, studies on the uracil nucleobase have shown that DFT calculations match well with more accurate ab initio methods.^42^

Building on this, we modeled the electron attachment process of NABs in the gas phase through DFT calculations at a reasonable computational cost. Subsequently, we applied a quantum mechanics/molecular mechanics (QM/MM) approach, the Perturbed Matrix Method (PMM),^43^ to ascertain the redox thermodynamics of nucleic acid units: the five nucleobases and their corresponding nucleosides in the condensed phase, explicitly taking into account the environment effects (e.g., solvent and ions). The same general hybrid approach has been successfully applied to model one-electron oxidation thermodynamics involving the same molecules.^44–46^

## Theory

The Perturbed Matrix Method (PMM) is a hybrid quantum/classical theoretical-computational method that shares similarities with other QM/MM approaches.^47–51^ In this section, we briefly summarize the general idea behind the method (for a more detailed description, the reader is referred to Theory section of the Supporting Information (SI)). The system of interest is divided into two regions: the quantum center (QC), the region of the system treated quantum mechanically—for which the properties of interest are calculated using electronic structure methods—and the surrounding environment, which acts as a semiclassical dynamical perturbation influencing the QC. Typically, the environmental configurations are obtained by all-atom classical MD simulations. The method follows an *a posteriori* strategy, assuming a semirigid QC. The electronic properties of the isolated QC (unperturbed properties) are calculated quantum chemically in vacuum for one or a few reference configurations, and for each configuration sampled by MD simulations of the whole system, the effect of the inhomogeneous electric field, from the instantaneous atomistic configurations of the environment, is included as a perturbing term within the QC Hamiltonian operator. By diagonalizing the perturbed electronic Hamiltonian matrix at each MD frame, a trajectory of the perturbed electronic eigenstates and energies are obtained. The eigenvalues and the eigenvectors can be used for evaluating the quantum observable of interest of the perturbed QC. In the present case, the QC ground state energy of the neutral and the radical anion forms of the NABs in their corresponding environments.

The Helmholtz free energy change, Δ*A*, associated with the reduction reaction, *B* + *e* ^–^ ⇌ *B* ^•–^, can be calculated according to

1 $$\Delta A≃\frac{k_{B}T}{2}\ln\frac{\langle e^{\beta \Delta U_{e}}\rangle_{\mathrm{red}}}{\langle e^{-\beta \Delta U_{e}}\rangle_{\mathrm{ox}}}$$

where *k*_B_ is the Boltzmann constant, β = (*k*_B_ *T*)^−1^, $\Delta U_{e}$ represents the energy change associated with the reduction of the generic species *B* (obtained by means of PMM-MD), and the angular brackets represent the average in the neutral (ox) or reduced (red) ensemble, respectively, each with its own ionic conditions. Further details on the derivation of eq 1 can be found in the Theory section of the SI. The PMM-MD approach has been successfully applied to model the redox properties of complex systems in the condensed phase.^44–46^ Finally, the reduction potential is calculated using the Nernst equation

2 $$E=-\frac{\Delta A}{\mathrm{nF}}$$

where *F* is the Faraday constant, Δ*A* is the Helmholtz free energy change, and *n* is the number of electrons involved in the reaction. All the reduction potential values are reported using the standard hydrogen electrode (SHE) as a reference (*E*_SHE_^⊖^ = 4.281 V).^52^

## Computational Methods

### Quantum Mechanical Calculations

The structures of the five NABs adenine, guanine, cytosine, thymine and uracil were optimized in the gas phase in their neutral and VB radical anion states at different levels of theory. The optimizations were carried out at the DFT level using the CAM-B3LYP^53^ functional with the 6-311++G­(2d,2p)^54,55^ basis set. The functional was found to be capable of reproducing the calculated gas-phase AEAs of the investigated nucleobases at more expensive levels of theory^17,18^ and the measured gas-phase AEA of the uracil and thymine nucleobases.^13,16^ The same gas-phase optimizations were also carried out using the M06-2X^56^ functional and 6-31++G­(d,p) basis and wave function-based methods such as Møller–Plesset to a second order (MP2) and Couple Cluster Singles and Doubles (CCSD), using the aug-cc-pVDZ^57^ basis set. Using both of the latter methods, the convergence to the minimum associated with the VB state of the adenine radical anion in gas phase was not achieved.

On top of the CAM-B3LYP/6-311++G­(2d,2p) ground state optimized structures, the unperturbed properties, i.e., energies, permanent and transition dipole moments, and ESP atomic charges,^58,59^ of the ground and the first four excited states were obtained by means of time-dependent DFT using the same functional and the same basis set. The unperturbed properties of the neutral and VB radical anion states of the five nucleobases are needed to apply the PMM-MD procedure (see Theory section and SI).

To estimate the VEAs of the nucleobases in gas phase, we performed single-point calculations at CAM-B3LYP/6-311++G­(2d,2p) level to obtain the energies of the radical anions at the geometry of the corresponding neutral minima. To compare our results with implicit solvent approaches, the five nucleobases were also optimized in water and DMF solvents, both modeled within the Polarizable Continuum Model using the integral equation formalism (IEFPCM),^60,61^ in their neutral and VB radical anion states.

Furthermore, the optimized geometries of the anti and syn conformers of the neutral and radical anion nucleosides of the five nucleobases were obtained at CAM-B3LYP/6-311++G­(2d,2p) level of theory in gas phase, in water and in DMF as solvents. Consistently, these were modeled using the IEFPCM model. The ESP atomic charges of the nucleosides (in both anti and syn conformations) were calculated to estimate the amount of localization of the negative charge on the nucleobase moiety. Each stationary point was confirmed to be a minimum by performing vibrational frequency calculations at the same level of theory, except for the CCSD optimizations. The Cartesian coordinates of these optimized structures are reported in the indicated repository. All electronic structure calculations were performed using the Gaussian 16 software package.^62^

### Molecular Dynamics Simulations

Several sets of MD simulations were performed to apply the PMM-MD method. The neutral and (valence-bound) radical anion forms of the adenine, guanine, cytosine, thymine, and uracil nucleobases were simulated in water and in DMF, providing the configurations of the neutral and radical anion ensemble, respectively.

Additionally, MD simulations of the neutral and radical anion forms of the corresponding nucleosides (adenosine, guanosine, cytidine, thymidine, and uridine) were performed in water and DMF to obtain the corresponding neutral and reduced ensemble. The General Amber Force Field (GAFF)^63^ parameters of the neutral nucleobases were obtained using the AnteChamber PYthon Parser interfacE (ACPYPE)^64^ software package, and the (ESP) charges calculated at CAM-B3LYP/6-311++G­(2d,2p) were integrated and used in the force field. The same procedure was used for the calculation of the atomic charges of the radical anion states (all the other force field parameters identical to those of the corresponding neutral form).

The remaining parameters were taken from the ParmBSC1 force field^65^ to model both the neutral and the reduced state of the five nucleosides. The GAFF parameters for the DMF solvent molecules were obtained consistently using the ACPYPE software package. The TIP3P model for the water solvent molecules was used.^66^

The same simulation protocol was used for all systems in both the redox states and for both types of solution. Each structure was placed in the center of a cubic box of ca. 3.0 and 4.5 nm for the simulations in water and DMF solutions, respectively, and solvated with ca. 900 water molecules and ca. 500 DMF molecules, respectively. In the case of radical anion states, one solvent molecule in the box was replaced by Na^+^ in water and tetrabutylammonium (TBA^+^) counterion in DMF to achieve electroneutrality of the system. The force field parameters for the TBA^+^ ion were obtained using the ACPYPE software package.

After an initial energy minimization, the entire system was gradually heated to the desired temperature of 300 K, using short (50 ps, with a time step of 2 fs) NVT MD simulations. The system was then equilibrated at 300 K with short (50 ps, with a time step of 2 fs) NVT MD runs, tuning the size of the box to reproduce the correct solvent bulk density at the temperature of 300 K and pressure of 1 bar. The temperature was kept constant using the V-rescale algorithm^67^ using a τ_*T*_ of 0.1 ps. The LINCS algorithm^68^ was used to constrain all covalent bonds involving a hydrogen atom. The electrostatic interactions were calculated using the particle mesh Ewald method^69,70^ with a cutoff radius of 1.1 nm. The same cutoff values were used for the van der Waals interactions. After the equilibration step, each system was simulated for 100 ns, using a time step of 2 fs, at fixed temperature and volume (NVT ensemble). All MD simulations were performed using the GROMACS 2022.4 software package.^71^

In the case of the nucleosides, the QC-environment partition splits the system along a chemical bond between the carbon atom of the ribose and nitrogen atom of the nucleobases (see Figure S1 in SI). Therefore, a hydrogen atom is introduced as a link atom^72^ at the QM/MM boundary to cap the QC subsystem. It is present in the QM calculations on the QCs, i.e., the NABs, but not in the MD simulations of the whole system.

The frames used in the MD-PMM procedure were evenly sampled from the MD simulations every 2 ps. In total, 50,000 MD frames were used in the QM/MM calculation.

## Results and Discussion

### Vertical and Adiabatic Electron Affinities in the Gas Phase

The VEAs obtained from the optimized geometries of the neutral and radical anion nucleobases in gas phase at CAM-B3LYP/6-311++G­(2d,2p) are reported in Table 1 and compared to the literature. The computed VEA values at the DFT level of theory are in line with values obtained using higher levels of theory and are rather close to the available experimental range of values. Although the energetics of the process is well captured, it is worth noting that the experimental values reported in Table 1 were interpreted as the VEA of the VB states of the radical anions of the nucleobases in gas phase while in the present work the radical anions at the Franck–Condon geometries of the neutral states were found to be DB in nature for all the five nucleobases (see Figures 1 and S2 in SI for the shape and spatial extension of the radical anions SOMOs), in agreement with Dutta et al.,^29^ for the adenine, guanine, and cytosine nucleobases, who employed the EOM-EA-CCSD level of theory. Moreover, as reported by other previous experimental works, slightly positive electron affinities were observed for the DB states of uracil and thymine.^24,25^

**Figure 1:**
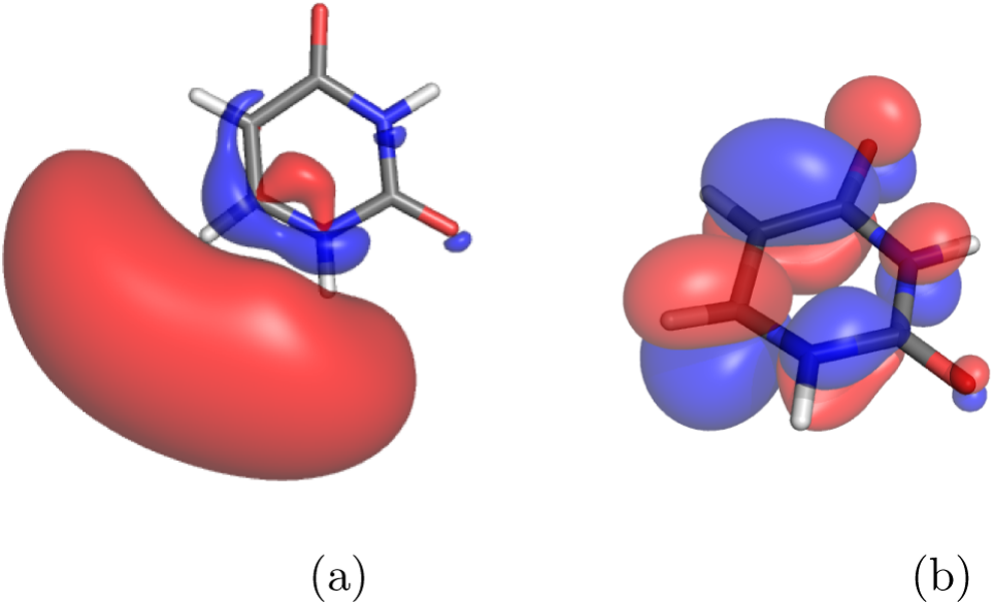
SOMO of the uracil radical anion at (a) the Franck–Condon geometry of the neutral state, and (b) the valence-bound radical anion optimized geometry, computed at CAM-B3LYP/6-311++G­(2d,2p) level of theory.

**Table 1:** Calculated and Experimental Vertical Electron Affinities (VEAs) in eV

reference	method	uracil	thymine	cytosine	guanine	adenine
Li et al.^27^	B3LYP/6-31G(d)	–1.09	–1.05	–1.42	–2.07	–1.57
	B3LYP/6-31+G(d)	–0.35	–0.29	–0.67	–0.41	–0.85
	B3LYP/6-311++G(2d,p)	–0.18	–0.22	–0.17	–0.07	–0.34
Roca–Sanjuán et al.^17^	MP2/aug-cc-pVDZ	–0.69	–0.73	–0.91	–1.57	–1.42
	PMP2//MP2/aug-cc-pVDZ	–0.56	–0.58	–0.73	–1.30	–0.99
	CCSD/aug-cc-pVDZ	–0.63	–0.65	–0.77		
	CCSD(T)//CCSD/aug-cc-pVDZ	–0.64	–0.65	–0.79		
	CASPT2/ANO-L 4321/321^a^	–0.49	–0.45	–0.59	–0.94	–0.74
Dutta et al.^29^	EOM-EA-CCSD/aug-cc-pVDZ^b^	–0.23	–0.28	–0.27	–0.23	–0.44
	EOM-EA-CCSD/aug-cc-pVTZ^b^,^c^	–0.20	–0.24	–0.24	–0.19	–0.40
present work	CAM-B3LYP/6-311++G(2d,2p)	–0.29	–0.33	–0.34	–0.29	–0.52
present work	M06-2X/6-31++G(d,p)	–0.49	–0.53	–0.76	–0.39	–0.67
experimental range^12,15,22^		–0.30 to −0.22	–0.53 to −0.29	–0.55 to −0.32		–0.56 to −0.45

a Using CASSCF/ANO-L 4321/321 optimized geometries.

b Using B2PLYP/aug-cc-pVTZ optimized geometries.

c The aug-cc-pVDZ basis was used for the hydrogen atoms.

The difficulties in simultaneous prediction of the correct nature of the anionic state (valence- or dipole-bound) and the vertical electron affinity of the nucleobases lie in the fact that the use of large and diffuse basis sets, crucial for the description of these anionic species,^73^ induces contamination of the VB state with the DB state, as previously observed.^27^ Thus, employing extended basis sets, the wave function of a valence anion, which is unbound at the equilibrium geometry of the neutral molecule, might result in a DB state. In the limit of an extremely diffuse basis set,^26^ the calculated VEA values should approach the slightly positive values experimentally found for the DB states.^24,25^ Indeed, for the uracil nucleobase, it was previously shown by Bachorz et al.^74^ that the use of the CCSD­(T) level of theory and the AVDZ basis set supplemented with additional diffuse functions centered on the H­(C6) atom leads to a positive AEA for the DB state of the uracil anion, with the latter showing an almost negligible structural reorganization with respect to the neutral state. The latter work agrees with the experimental observations that the DB anion of uracil is the dominant form in the gas phase and with the lack of a significant Franck–Condon progression in the photoelectron spectrum of the anion in the gas phase.^30^

Upon solvation or microsolvation, the electronic capture ability of the nucleobases increases.^3,75^ Both dipole- and valence-bound states of microhydrated thymine and cytosine were found to be energetically favored by the presence of explicit water molecules with respect to the gas phase.^76,77^ The same trend was found for the VB anions of the uracil-water clusters.^78,79^ Finally, an energetically favored DB state was found for the adenine-water clusters.^80^ Moreover, it was shown that upon solvation or microsolvation, the DB state of uracil collapses into a VB state as suggested by photoelectron measurements on the uracil anion solvated by one water molecule^30^ and by static calculations on uracil-water complexes^78^ that match well the previously cited photoelectron spectra. Lastly, an initial anionic state with a delocalized electron density over the base and several water solvent molecules tends to rapidly localize (on the femtosecond to picosecond time scale) on the solute, as shown by ab initio molecular dynamics simulations of the four DNA nucleobases in water.^19,75,81^ Therefore, this work focuses on the description of the VB states because they are the relevant anionic states in solution and biological media. For a more in-depth discussion of this topic, the reader is referred to available literature.^3,5^

Our initial effort was directed to locate the minima associated with the VB states of the radical anions in the gas phase. To confirm the VB nature of the electron-attached state, the SOMOs (reported in Figures 1 and S3 in SI) were inspected and it was found that the electron occupies one of the π* orbitals of the nucleobase. Such geometries were then used to calculate the corresponding unperturbed properties (i.e., energies, dipole moments, and ESP atomic charges) needed to apply the PMM-MD method to estimate the redox thermodynamics of the nucleobases and nucleosides in solution. As described in the Computational Methods section, we optimized the neutral and radical anions of the nucleobases with DFT and wave function-based methods to calculate the AEAs reported in Table 2. From our estimates and the DFT-based calculations available in the literature, a clear effect of the basis set emerges. Moreover, it was previously suggested that, within the CCSD level of theory, the aug-cc-pVDZ basis set is still too small to correctly describe the VB anion of uracil.^28^ The CAM-B3LYP functional in conjunction with the 6-311++G­(2d,2p) basis set results in a satisfactory agreement with the experimental data available for the pyrimidine nucleobases and therefore it was selected for further calculations due to the compromise between accuracy and computational cost. The convergence of the AEA, estimated with CAM-B3LYP, with respect to the basis set size was investigated for the uracil nucleobase (see Figure S4 in SI). The AEA associated with the VB state is not dramatically affected by the basis set size.

**Table 2:** Calculated and Experimental Adiabatic Electron Affinities (AEAs) in eV

reference	method	uracil	thymine	cytosine	guanine	adenine
Russo et al.^35^	B3LYP/D95++**	0.23	0.19	0.02	0.00(1)	–0.26
Li et al.^27^	B3LYP/6-31G(d)	–0.52	–0.49	–0.69	–1.51	–1.18
	B3LYP/6-31+G(d)	0.18	0.20	–0.06	–0.32	–0.40
	B3LYP/6-311++G(2d,p)	0.20	0.22	–0.05	–0.30	–0.01
Lewis et al.^18^	M06-2X/6-31++G(d,p)		–0.09	–0.24	–0.34	–0.72
Roca-Sanjuán et al.^17^	MP2/aug-cc-pVDZ	–0.21	–0.26	–0.40	–0.71	–1.06
	PMP2//MP2/aug-cc-pVDZ	–0.09	–0.14	–0.25	–0.63	–0.88
	CCSD/aug-cc-pVDZ	–0.07	–0.12	–0.18	–0.50	–0.90
	CCSD(T)//CCSD/aug-cc-pVDZ	–0.05	–0.09	–0.17	–0.44	–0.84
	CASPT2/ANO-L 4321/321^a^	0.10	0.19	0.08	–0.20	–0.39
Dutta et al.^29^	EOM-EA-CCSD/aug-cc-pVDZ	–0.13	–0.19	–0.24	–0.15	–0.41
	EOM-EA-CCSD/aug-cc-pVTZ^b^,^c^	–0.15	–0.20	–0.21	–0.12	–0.38
present work	CAM-B3LYP/6-311++G(2d,2p)	0.18	0.14	–0.03	–0.30	–0.48
present work	M06-2X/6-31++G(d,p)	0.08	0.04	–0.14	–0.38	–0.58
present work	MP2/aug-cc-pVDZ	–0.09	–0.14	–0.25	–0.63	^e^
present work	CCSD/aug-cc-pVDZ^d^	–0.07	–0.12	–0.18	–0.49	^e^
experimental^13^		0.15 ± 0.12	0.12 ± 0.12	0.13 ± 0.12		

a On top of the CASSCF/ANO-L 4321/321 optimized geometries.

b On top of the B2PLYP/aug-cc-pVTZ optimized geometries.

c The aug-cc-pVDZ basis was used for the hydrogen atoms.

d ZPE corrected values at MP2/aug-cc-pVDZ.

e The valence-bound minimum was not found at this level of theory.

### Neutral and Anion Geometries

In this section, we analyze the main geometrical differences between the equilibrium geometries of the neutral and (VB) radical anion nucleobases. The valence anion geometries are accompanied by modest structural changes with respect to the neutral forms. As reported in Figure 2a (left) and b, the pyrimidine ring of uracil, thymine, and cytosine adopts a nonplanar geometry and the methyl group of thymine rotates with respect to the corresponding neutral structure. The rotation of the methyl group in the thymine radical anion is responsible for the relatively high root-mean-square deviation (RMSD) with respect to the other pyrimidine base anions. Similarly, the five-membered ring of the adenine nucleobase undergoes out-of-plane distortion after the electron attachment, thus showing a similar RMSD displayed by uracil and cytosine bases. The guanine base shows the highest structural change, explained by the loss of planarity of the six-membered ring and, contextually, by the rotation of its amino group. Some of these findings are consistent with previous structural analyses present in the literature.^17,29^ Of note, the CAM-B3LYP geometries of both the neutral and radical anion of the nucleobases display the same geometrical features observed at the CCSD level of theory and the RMSD between the two sets of geometries is always lower than 0.04 Å, except for the thymine anion. The slightly higher deviation for the latter anionic base is due to the different degrees of rotation of the methyl group provided by the two levels of theory. Nevertheless, an overall agreement among the CAM-B3LYP functional and CCSD was found in terms of geometries.

**Figure 2:**
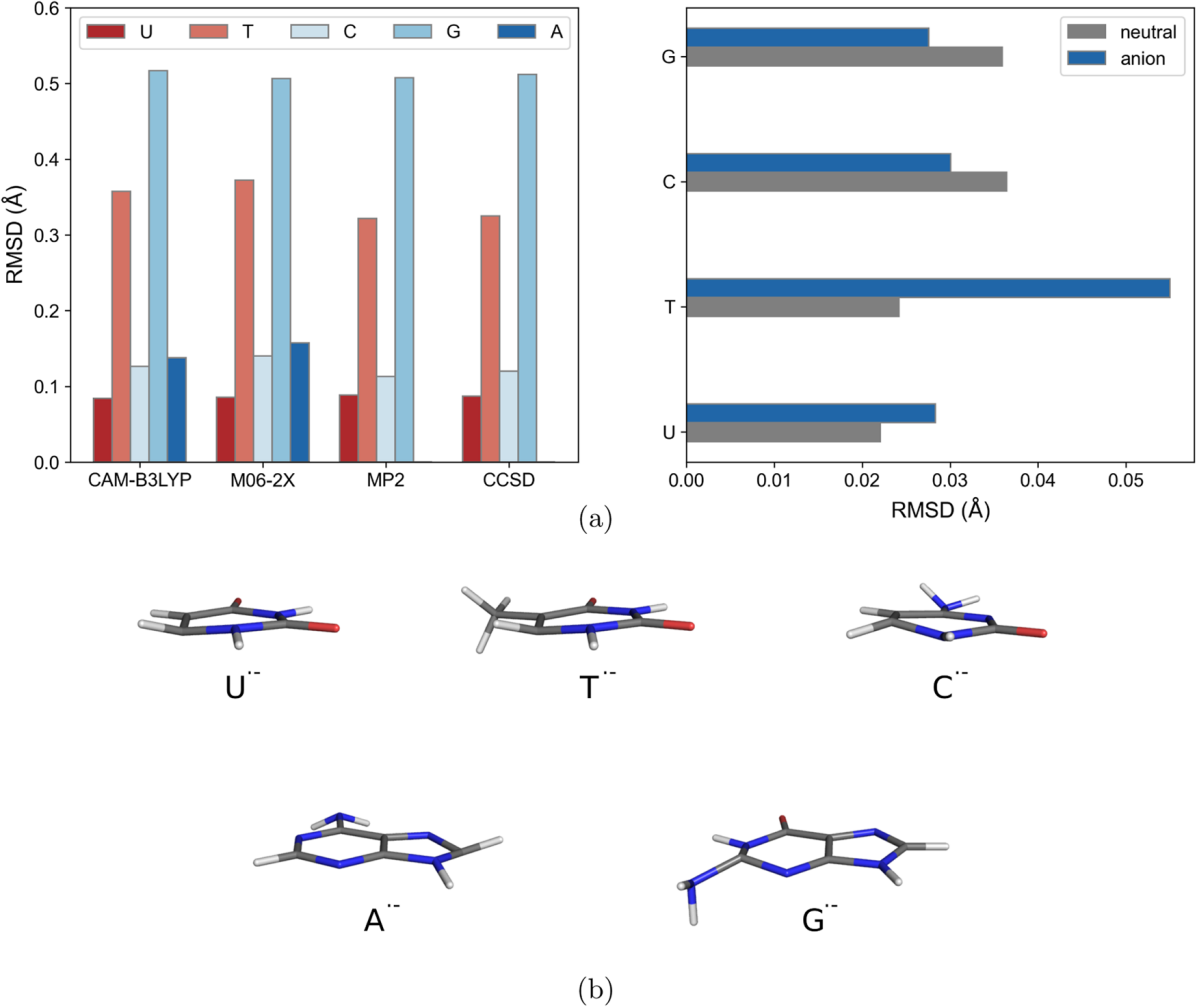
(a) Left: Root mean square deviation (RMSD) of the (valence-bound) radical anion with respect to the neutral equilibrium structure of the five nucleobases at different levels of theory. Right: Root mean square deviation (RMSD) of the equilibrium structures obtained at CAM-B3LYP with respect to the ones obtained at CCSD level of theory. (b) Optimized geometries of the radical anions at CAM-B3LYP level of theory.

Building on this, we optimized the structures of the neutral and the valence bound radical anion states of all the nucleosides. All the optimizations converged to a minimum as confirmed by the frequency analysis. The nucleobase moiety of the corresponding nucleoside radical anion, in both syn and anti conformations, retains similar structural features observed in the VB anion geometry of the base alone, as suggested by the RMSD that never exceeds 0.35 Å for all the compounds, as shown in Figure S5. Similar results were obtained in the presence of solvents (i.e., water and DMF) described at the PCM level of theory.

### Adiabatic Electron Affinities and Reduction Potentials in Solution

The reduction potentials of all five bases and their respective nucleosides in water and DMF were calculated using the PMM-MD approach. For all systems, the nucleobase was chosen as the QC and thus treated at the quantum mechanical level (CAM-B3LYP/6-311++G­(2d,2p)). To support the selection of the nucleobase as QC for the nucleoside systems, we calculated the ESP atomic charges of the nucleoside radical anions in gas phase, water, and DMF, as explained in the Computational Methods section. The analysis of the atomic charges, reported in Table S1 in SI, shows that the negative charge in the reduced species (in both anti and syn conformers) is localized on the nucleobase moiety. The presence of the continuum dielectric enhances the localization of the charge on the nucleobase, supporting the choice of the nucleobase as the QC.

As reported in the Theory section, to estimate the reduction potentials, it is necessary to calculate the perturbed energies of the nucleobases and nucleosides in both the neutral and reduced ensembles. First, we estimated the AEA for all species studied in solution as the mean perturbed energy difference between the two corresponding redox states in the neutral and charged ensemble. The results, reported in Table 3, indicate a shift toward more positive values for all bases in water and in DMF, with respect to the gas phase, according to previous works.^21,31,36–38^ Such an effect is due to the stabilizing effect of the polar solvent on the reduced species. Furthermore, the AEA of the nucleobases and the corresponding nucleosides in both anti and syn conformations in the gas phase, water, and DMF (the latter two modeled using PCM) were calculated at CAM-B3LYP/6-311++G­(2d,2p) level. The results are in line with those obtained through the PMM-MD approach (see Tables S2 and S3 in SI).

**Table 3:** Calculated Adiabatic Electron Affinity (AEA) of the Nucleobases and Nucleosides in Gas Phase, Water, and DMF in eV^a^

AEA (eV)							
	nucleobase				nucleoside		
	gas-phase	water	DMF		gas-phase^b^	water	DMF
U	0.18	2.52	1.75	Urd	0.68	2.80	2.05
T	0.14	2.35	1.68	Thd	0.63	2.62	1.95
C	–0.03	2.28	1.52	Cyd	0.50	2.61	1.94
G	–0.30	1.83	1.08	Guo	0.37	2.13	1.45
A	–0.48	1.55	1.08	Ado	0.13	2.11	1.53

a The estimated standard error on the calculated AEAs in the condensed phase is ±0.04 eV.

b Anti conformations.

In addition, we carried out the analysis of the perturbation—as provided by the inhomogeneous electric field generated by the dynamical environment—on the QCs (the NABs) in the two redox states. The effect of the environment was estimated as the difference between the ensemble average of the perturbed electronic energy and the gas-phase electronic energy, i.e., $\Delta_{\mathrm{rdx}}^{\mathrm{ens}}={\left\langle U_{e,\mathrm{rdx}}\right\rangle }_{\mathrm{ens}}-U_{e,\mathrm{rdx}}^{0}$ , with rdx indicating the redox state of the QC, and the subscript ens indicating the ensemble in which the average is performed. The estimates of such properties, reported in Table S4 in SI, show that the stabilizing contribution of the perturbation is between 0.4 and 2.5 eV for all the species in both water and DMF, except for the reduced (radical anion) states in their own (ionic) environment. In fact, the perturbation of these anions in their reduced ensemble is more pronounced, with a stabilizing effect in the range of 4–5 eV in DMF and up to 7.5 eV in water. The effect of the sugar moiety results in an additional stabilizing effect of up to 0.5 eV for the reduced form of the nucleosides in the anionic ensemble compared to the corresponding nucleobases. This is reflected in the AEA values of the nucleosides, which are more positive by ca. 0.2–0.3 eV with respect to the corresponding NABs (see Table 3 and Figure 3a and b). Notably, a similar shift is also evident when the whole nucleosides are treated quantum mechanically using PCM as a solvation model (see Table S3 in SI). To ensure that our sampling is suitable for modeling these observables, we verified the convergence of the reduction free energy, Δ*A*, (see eq S11 in SI) and of the condensed-phase AEAs. As shown in Figures 3 and S6, all calculated AEAs and Δ*A* converge reasonably within the simulation time.

**Figure 3:**
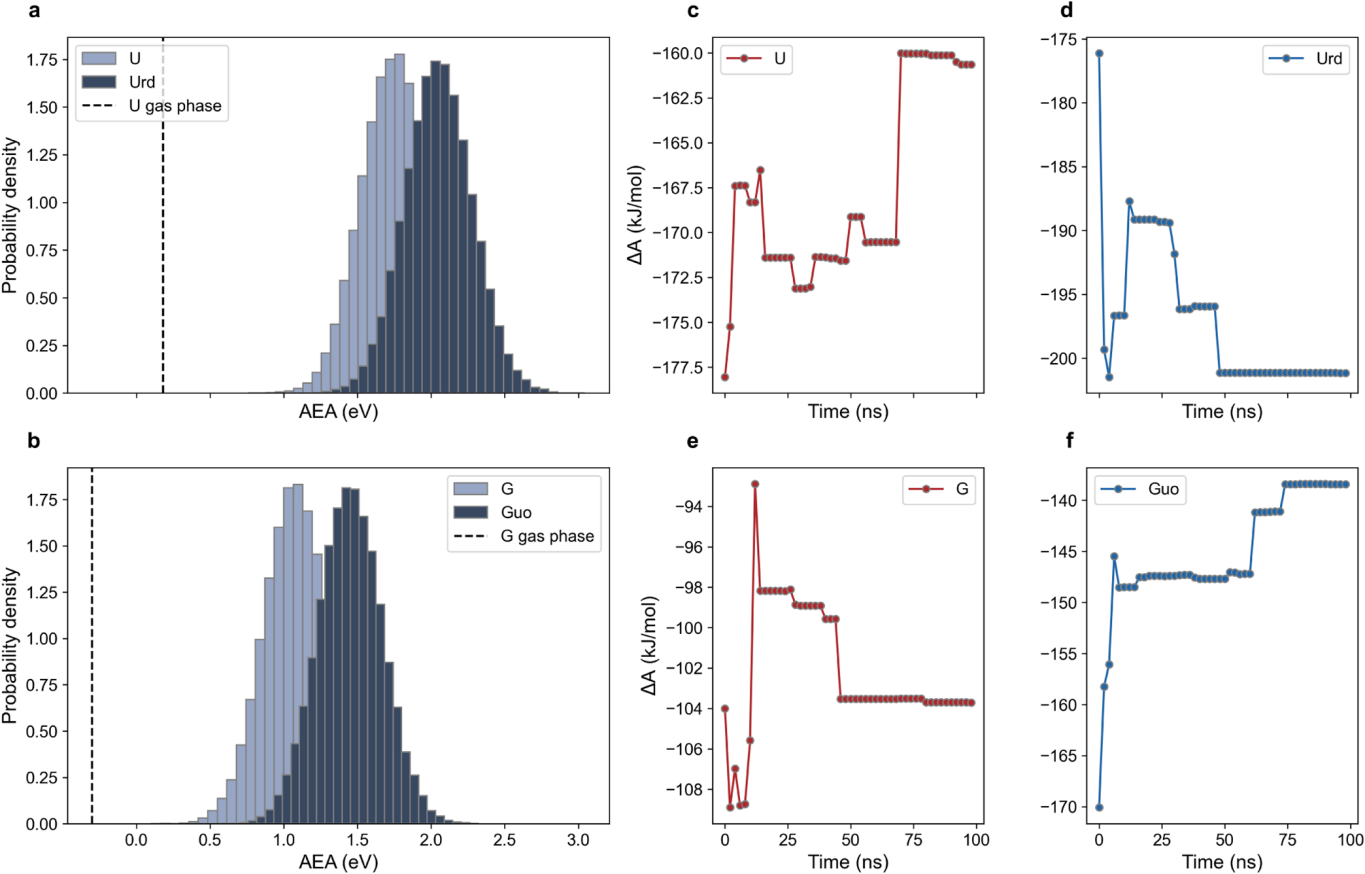
Convergence of the redox properties for uracil (U), uridine (Urd), guanine (G), and guanosine (Guo) in solution. (a) AEA distributions for uracil and its nucleoside, and (b) guanine and its nucleoside in DMF (AEA gas-phase value as a dashed vertical line). Helmholtz free energy change calculated as a function of the simulation time for the reduction of (c) uracil, (d) uridine, (e) guanine, and (f) guanosine in DMF.

The five nucleosides were first studied in DMF to make a tight comparison with the reduction potentials (vs SHE) measured experimentally.^11^ The results, reported in Table 4, show satisfactory agreement with the experimental data, with deviations always below 0.4 V for all the molecules analyzed. Moreover, the reduction potentials were obtained by means of the same PMM-MD scheme modeling the QC at the CCSD/aug-cc-pVDZ level of theory (see Table S5). However, as previously noted, this approach does not describe the process as effectively as CAM-B3LYP/6-311++G­(2d,2p), probably because of the aug-cc-pVDZ basis set that is still not sufficiently large. The effect of the presence of ribose was then investigated by calculating the reduction potentials of the nucleobases. According to our estimates, the sugar moiety stabilizes the reduced species (see Table S4), resulting in a more negative potential of approximately 0.5 V for all the NABs with respect to the corresponding nucleosides. Such a result is in line with our previously obtained values of the AEAs in DMF for all the studied species (see Tables 3 and S3). The stabilizing effect of the sugar moieties is maintained in the aqueous environment, consistent with the results shown in Tables 3 and S3. Furthermore, our estimates indicate that the reduction potentials of thymine and cytosine nucleobases and nucleosides are almost indistinguishable within the experimental-computational error, in both solvents. This finding suggests that the redox behavior of these bases is almost identical to that predicted by our computational procedure and reported in the literature.^11^ Finally, the estimated values of the reduction potentials in water indicate a positive shift of this observable for all of the systems, thus confirming that the aqueous environment promotes the reduction of both nucleobases and nucleosides with respect to the gas phase and the DMF solution.

**Table 4:** Calculated Reduction Potential of the Nucleobases and Nucleosides in Water and DMF in V^a^

*E* (V)						
	nucleobase			nucleoside		
	water	DMF		water	DMF	exp.^11^
U	–1.65	–2.59	Urd	–1.50	–2.19	–2.07
T	–2.05	–2.63	Thd	–1.66	–2.31	–2.14
C	–1.93	–2.85	Cyd	–1.60	–2.27	–2.23
G	–2.43	–3.23	Guo	–2.16	–2.91	<−2.76
A	–2.72	–3.19	Ado	–2.12	–2.77	–2.45

a Values are reported against SHE.^52^ The estimated standard error on the calculated reduction potentials is ±0.07 V.

## Conclusions

Combining all-atom MD simulations and reliable QM electronic properties, we employed the PMM-MD approach to estimate the AEAs and reduction potentials of nucleic acid components in water and DMF solutions. Our calculations, in good agreement with the available experimental data (only available in DMF), indicate that the sugar moiety present in the nucleosides and the solvent, in particular water because of its high polarity, stabilize the radical anion form of the NABs. Such an effect is reflected in the increased values of the AEAs and redox potentials obtained for all the species in solution compared to those of the gas phase. The same behavior is observed for the nucleosides, where our estimates of the same observables are less negative (or more positive) than those of the corresponding bases. To the best of our knowledge, we report one of the first systematic computational estimates of the reduction potential (or equivalently free energy differences) of nucleic acid building blocks by explicitly accounting for complex environments, including water, DMF, and ions. Furthermore, our results provide a solid foundation for modeling the thermodynamics of electron attachment in more complex systems, such as single and double DNA/RNA strands or other biologically relevant molecules.

## Supplementary Material



## Data Availability

All optimized nucleobases and nucleoside geometries obtained in this work are openly available at the following Zenodo repository.

## Notes

Detailed explanation of the theory behind the PMM method and how the free energy differences are estimated. QM/MM boundary and the link atom; SOMOs of the DB and VB radical anions of the NABs obtained at the CAM-B3LYP/6-311++G­(2d,2p) level of theory; dependency of the AEA (estimated using DFT and CAM-B3LYP as a functional) on the basis set size. Effect of the sugar moiety on the nucleobase geometries. Charge distributions of the nucleosides radical anions, the AEAs of the nucleobases and nucleosides in the gas phase, water, and DMF; Convergence of the AEA as a function of the simulation time. Analysis of the environmental electrostatic perturbation from the PMM-MD calculations. Reduction potentials of nucleosides at the CCSD level of theory (PDF)
