# Supplementary material for: Theoretical Modeling of the Redox Thermodynamics of Nucleic Acid Building Blocks in the Condensed Phase
Source: J Phys Chem B. 2025 Aug 29;129(38):9578–87. doi: 10.1021/acs.jpcb.5c03817 (PMC12478868; doi:10.1021/acs.jpcb.5c03817)
Supplement: Supplementary file 1 [file jp5c03817_si_001.pdf]

Supporting Information: Theoretical Modeling of the Redox Thermodynamics of Nucleic Acids Building Blocks in the Condensed Phase

Alessio Olivieri,[†] Alessandro Nicola Nardi,[*,‡] and Marco D'Abramo[*,†]

†*Department of Chemistry, Sapienza University of Rome, 00185 Rome, Italy*

‡*Nantes Université, CNRS, CEISAM UMR 6230, F-44000 Nantes, France*

E-mail: alessandro.nardi@univ-nantes.fr; marco.dabramo@uniroma1.it

Theory

Perturbed Matrix Method

The Perturbed Matrix Method (PMM)[1–3] is a hybrid quantum/classical computational method. Similarly to other QM/MM approaches, it assumes that the system can be divided into two distinct regions: one where the key quantum process occurs, the quantum center (QC), and the dynamical surrounding environment that represents an electrostatic perturbation to the QC. Assuming a semi-rigid QC, its unperturbed electronic properties (i.e., electronic ground and excited state energies, dipole moments, and atomic ESP charges) are obtained in vacuum at the desired ab initio level of theory for only one or a few representative configurations. The perturbation is provided by the inhomogeneous electric field arising from instantaneous configurations of the surrounding environment sampled via classical all-atom molecular dynamics (MD) simulations of the whole system. Therefore, including the electrostatic effect of the environment as a perturbing term on the QC, the perturbed Hamiltonian matrix, $\tilde{H}$, reads

$$\tilde{H} = \tilde{H}^0 + \tilde{V} \tag{S1}$$

where $\tilde{H}^0$ is the QC unperturbed (isolated) electronic Hamiltonian and $\tilde{V}$ is the perturbation matrix modeling the interaction between the QC and the surrounding environment. The elements $\left[\tilde{H}\right]_{l,l'}$ of the Hamiltonian matrix $\tilde{H}$ are conveniently expressed in the basis of the unperturbed electronic eigenstates, $\{\Phi^0\}_l$, by

$$\left[\tilde{H}\right]_{l,l'} = \langle \Phi_l^0 | \hat{H} | \Phi_{l'}^0 \rangle = \mathcal{U}_l^0 \delta_{l,l'} + \langle \Phi_l^0 | \hat{V} | \Phi_{l'}^0 \rangle \tag{S2}$$

where $\mathcal{U}_l^0$ is the unperturbed electronic energy of the l-th electronic state of the QC, and $\delta_{l,l'}$ is the Kronecker delta. The perturbation term can be expanded within the dipolar approximation

$$\hat{V} = \sum_j q_j \left[\mathcal{V}(\mathbf{r}_0) - \mathbf{E}(\mathbf{r}_0) \cdot (\mathbf{r}_j - \mathbf{r}_0) \right] \tag{S3}$$

to obtain the matrix elements $\left[\tilde{V}\right]_{l,l'}$ as follows

$$\left[\tilde{V}\right]_{l,l'} = \langle \Phi_l^0 | \hat{V} | \Phi_{l'}^0 \rangle = \mathcal{V}(\mathbf{r}_0) q_T \delta_{l,l'} - \mathbf{E}(\mathbf{r}_0) \cdot \langle \Phi_l^0 | \hat{\boldsymbol{\mu}} | \Phi_{l'}^0 \rangle \tag{S4}$$

where j runs over the QC particles (i.e., nuclei and electrons), q_j the charge of the j-th particle, $\mathbf{r}_j$ the corresponding coordinates; $\mathbf{r}_0$ is the position of the center of mass of the QC, $\mathcal{V}$ and $\mathbf{E}$ are the electrostatic potential and the perturbing electric field exerted by the classical environment, respectively, q_T is the total charge of the QC, and $\hat{\boldsymbol{\mu}}$ is the electric dipole moment operator. In typical PMM-MD calculations, the perturbing electric field is provided by the environmental atomic charges. Additionally, the perturbation operator $\hat{V}$ can be expanded within each N-th atomic region around the corresponding atomic center $\mathbf{R}_N$ (i.e., the nucleus position of the N-th atom of the QC), providing

$$\hat{V} = \sum_N \sum_j \Omega_N(\mathbf{r}_j) q_j \left[\mathcal{V}(\mathbf{R}_N) - \mathbf{E}(\mathbf{R}_N) \cdot (\mathbf{r}_j - \mathbf{R}_N) \right] \tag{S5}$$

with j running over all QC nuclei and electrons, N running over all QC atoms, and Ω_N a step function being null outside and unity inside the Nth atomic region. This atom-based expansion[3] is performed only for the diagonal elements; all other Hamiltonian matrix elements are calculated using a QC-based expansion within the dipole approximation (i.e., using eq. S4 to build the perturbed Hamiltonian matrix, $\tilde{H}$). Thus, from the latter expressions of the perturbation operator, the matrix elements $\left[\tilde{H}\right]_{l,l'}$ can be obtained as follows

$$\left[\tilde{H}\right]_{l,l'} = \left[\mathcal{U}_l^0 + \sum_N \mathcal{V}(\mathbf{R}_N) \langle \Phi_l^0 | \hat{q}_N | \Phi_{l'}^0 \rangle \right] \delta_{l,l'} - \mathbf{E}(\mathbf{r}_0) \cdot \langle \Phi_l^0 | \hat{\boldsymbol{\mu}} | \Phi_{l'}^0 \rangle (1 - \delta_{l,l'}) \tag{S6}$$

The derivation and discussion of the different flavors of the PMM approach can be found in a previous work in which the impact of the approximations involved in the PMM-MD

strategy are addressed.[3]

Helmholtz Free Energy and Redox Potential Calculation

The standard reduction potentials can be obtained through the Helmholtz free energy, according to the Nernst equation,

$$E = -\frac{\Delta A}{nF} \tag{S7}$$

where F is the Faraday constant and n is the number of electrons involved in the reaction. All the reported reduction potential values are obtained using the standard hydrogen electrode as reference ($E^{\ominus}_{SHE} = 4.281$ V).[4] The associated Helmholtz free energy change, ΔA, was estimated through

$$\Delta A = -k_B T \ln\langle e^{-\beta\Delta\mathcal{H}}\rangle_{ox} + \Delta A^{ion}_{red} = k_B T \ln\langle e^{\beta\Delta\mathcal{H}}\rangle_{red} + \Delta A^{ion}_{ox} \tag{S8}$$

where k_B is the Boltzmann constant, $\beta = (k_B T)^{-1}$ and $\Delta\mathcal{H}$ is the QC environment total energy change upon reduction. The averages, represented by angular parentheses, are evaluated either in the neutral (ox) or reduced (red) ensemble (i.e., QC is either in its reduced or oxidized state), accordingly to the angle bracket subscripts. Finally, ΔA^{ion}_{red} and ΔA^{ion}_{ox} are the relaxation free energy for the reduced species due to the $ox \rightarrow red$ ionic environment transition and the corresponding relaxation free energy for the oxidized species due to the $red \rightarrow ox$ ionic environment transition, respectively. Taking into account that the electronic state of the environment is nearly unaffected by the QC oxidation state, $\Delta\mathcal{H}$ can be approximated by the QC electronic energy difference, $\Delta\mathcal{U}_e$, between the energy minima associated with the quantum vibrational coordinates of the two states

$$\Delta A \simeq -k_B T \ln\langle e^{-\beta\Delta\mathcal{U}_e}\rangle_{ox} + \Delta A^{ion}_{red} = k_B T \ln\langle e^{\beta\Delta\mathcal{U}_e}\rangle_{red} + \Delta A^{ion}_{ox} \tag{S9}$$

Assuming $\Delta A^{ion}_{red} \simeq \Delta A^{ion}_{ox}$, the Helmholtz free energy change can be approximated

$$\Delta A \simeq \frac{k_B T}{2} \ln \frac{\langle e^{\beta \Delta \mathcal{U}_e} \rangle_{red}}{\langle e^{-\beta \Delta \mathcal{U}_e} \rangle_{ox}} \tag{S10}$$

In practice, the electronic energy change can be calculated via the PMM-MD method and the ensemble averages in the condensed phase performed over the corresponding MD sampling. The statistical errors were estimates by calculating the mean values of the Helmholtz free energy change in different sub-parts of the trajectory and evaluating the standard error. The convergence of the reduction free energy (ΔA) was checked by calculating ΔA as a function of the number of MD trajectory frames used, by

$$\begin{aligned} \Delta A(n) \simeq \frac{k_B T}{2} \ln \frac{\langle e^{\beta \Delta \mathcal{U}_e} \rangle_{red}}{\langle e^{-\beta \Delta \mathcal{U}_e} \rangle_{ox}} &= \frac{k_B T}{2} \ln \frac{\sum_{i=1}^{n} e^{\beta \Delta \mathcal{U}_e^{red}(n)}}{\sum_{i=1}^{n} e^{-\beta \Delta \mathcal{U}_e^{ox}(n)}} \\ &= \frac{1}{2} \left[k_B T \ln \sum_{i=1}^{n} e^{\beta \Delta \mathcal{U}_e^{red}(n)} - k_B T \ln \sum_{i=1}^{n} e^{-\beta \Delta \mathcal{U}_e^{ox}(n)} \right] \end{aligned} \tag{S11}$$

where i runs over the MD frames, in total n in number. The notation $\Delta \mathcal{U}_e^{ox}$ and $\Delta \mathcal{U}_e^{red}$ indicate the perturbed electronic energy difference in the sense of the reduction in the ensemble of neutral and radical anion, respectively.

In the present work, we simulated all the considered systems in their corresponding NVT ensembles. Consistent with this choice, the Helmholtz free energy was employed to address the thermodynamics of the redox processes.

QM/MM boundary and link atom

In the case of the nucleosides, the QC-environment partition splits the system along a chemical bond between the carbon atom of the ribose and nitrogen atom of the nucleobases. Therefore, a hydrogen atom is introduced as a link atom[5] at the QM/MM boundary to cap the QC subsystem. To illustrate this situation, we pictorially report the QM/MM boundary in Figure S1 for uridine. In the picture, the link atom (la) is a hydrogen atom which is present in the QM calculations on the QCs, i.e., the NABs, but not in the MD simulations of the whole system.

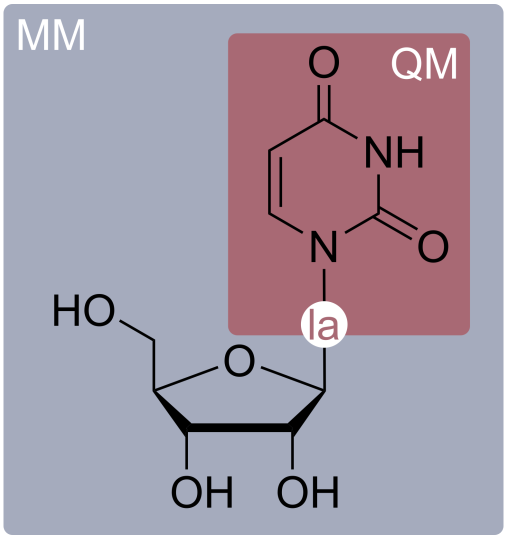

Figure S1: QM/MM partition of the system for the uridine nucleoside. The same scheme was adopted for the other nucleosides.

SOMOs of the radical anions of the nucleobases

SOMOs at the Franck-Condon geometries of the neutral states

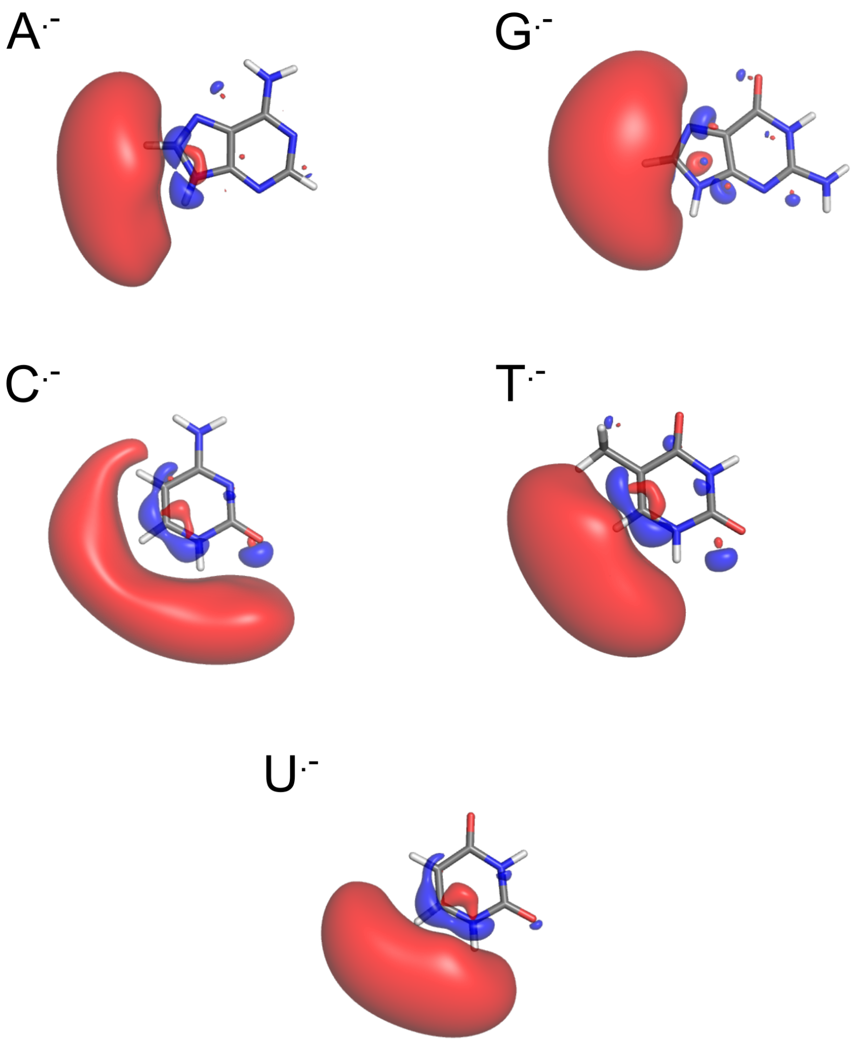

Figure S2: SOMOs of the radical anions of the nucleobases at the optimized geometries of the neutral states at CAM-B3LYP/6-311++G(2d,2p) level. Note the very diffuse nature of the SOMOs of the radical anions and their spatial extent outside the molecular region, indicating the dipole-bound nature of the anion in this region of the potential energy surface.

SOMOs at the valence-bound radical anion equilibrium geometry

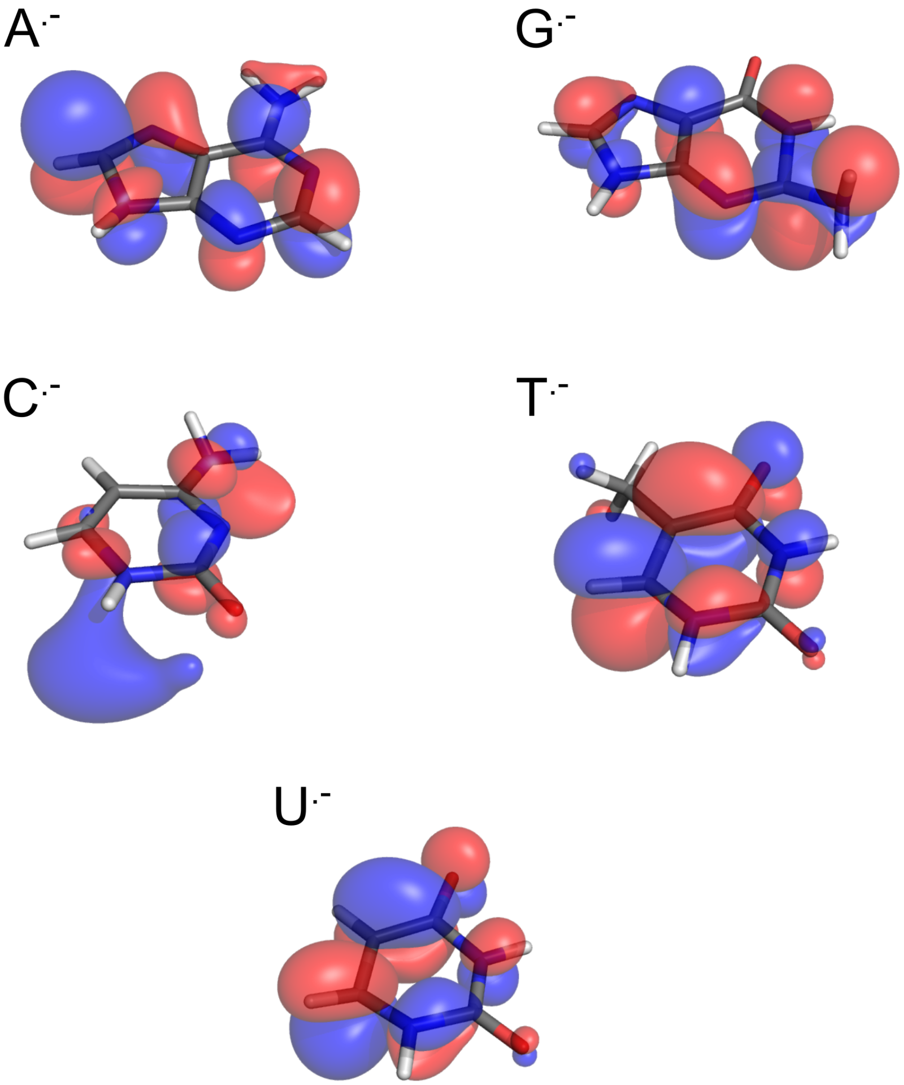

Figure S3: SOMOs of the valence-bound radical anions of the nucleobases at CAM-B3LYP/6-311++G(2d,2p) level. In the optimized geometry of the anion, the attached electrons occupies one of the π^* orbitals of the nucleobase.

Basis set size dependency of the AEA

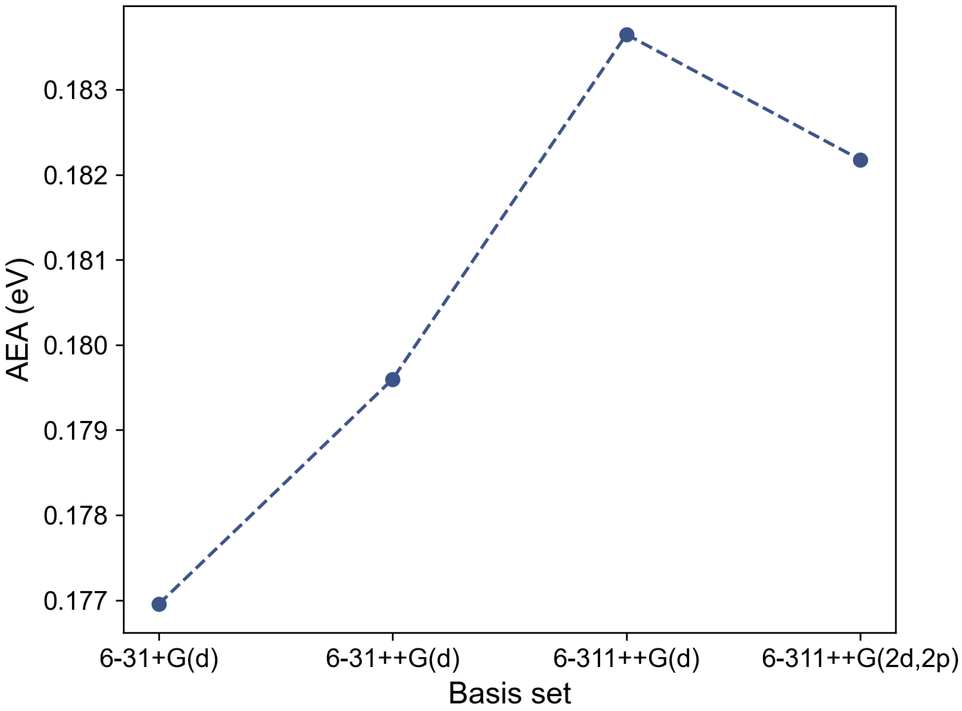

Figure S4: Adiabatic electron affinity (AEA) of the uracil nucleobase, taken as a test case, as a function of the basis set size. For each basis, the CAM-B3LYP functional was used and the geometries of both the neutral and the anion were optimized. The radical anion state is VB in nature.

Effect of the sugar moiety on the nucleobase geometries

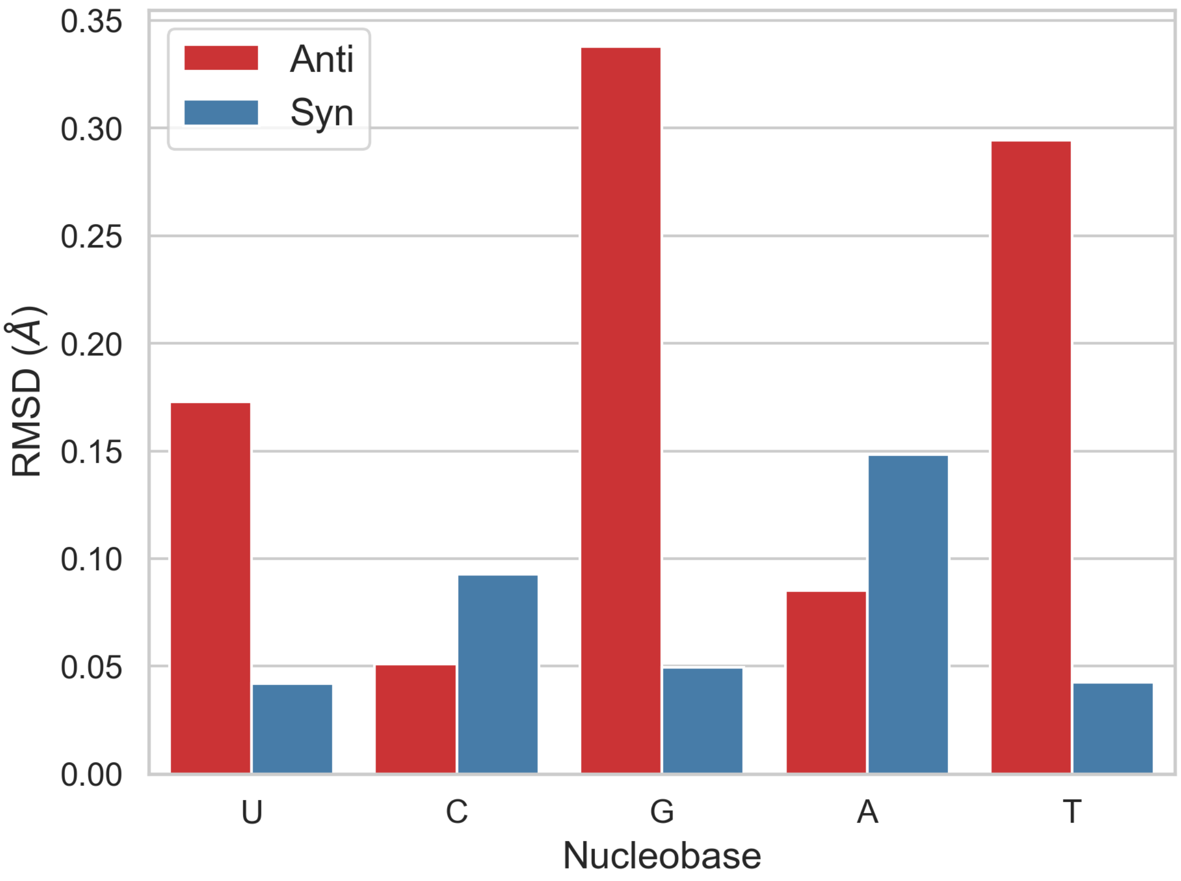

Figure S5: Root mean square deviation (RMSD) of the NAB moiety in the VB radical anion of the corresponding nucleoside (in both anti and syn conformations) with respect to the VB structure of the same nucleobase at the CAM-B3LYP level of theory in the gas phase.

Charge distribution in the radical anion of the nucleosides in gas phase, water and DMF (PCM)

Table S1: Distribution of the negative charge (a.u.) on the nucleobase in the corresponding nucleoside at the CAM-B3LYP/6-311++G(2d,2p) level of theory in gas phase, water and DMF (as modeled by PCM). The charge of the nucleobase fragment was obtained from the ESP[6,7] procedure on the whole nucleoside.

Nucleoside	Gas Phase		Water		DMF	
	Anti	Syn	Anti	Syn	Anti	Syn
Urd	-0.98	-1.04	-1.04	-1.10	-1.04	-1.11
Thd	-1.05	-1.10	-1.10	-1.00	-1.10	-1.12
Cyd	-0.91	-0.99	-0.97	-1.15	-0.97	-1.00
Guo	-1.06	-0.94	-0.92	-0.97	-0.92	-0.93
Ado	-0.83	-0.95	-1.04	-0.99	-1.03	-0.98

AEA of the nucleobases in gas phase, water and DMF (PCM)

Table S2: Calculated adiabatic electron affinities (AEAs) of bases at the CAM-B3LYP/6-311++G(2d,2p) level of theory in gas phase, water, and DMF (modeled by PCM) in eV.

Base	AEA (eV)		
	Gas Phase	Water	DMF
U	0.18	2.16	2.13
T	0.14	2.10	2.04
C	-0.03	2.00	1.97
G	-0.30	1.50	1.48
A	-0.48	1.59	1.55

AEA of the nucleosides in gas phase, water and DMF (PCM)

Table S3: Calculated adiabatic electron affinities (AEAs) of nucleosides at the CAM-B3LYP/6-311++G(2d,2p) level of theory in gas phase, water, and DMF (modeled by PCM) in eV.

Nucleoside	AEA (eV)					
	Gas Phase		Water		DMF	
	Anti	Syn	Anti	Syn	Anti	Syn
Urd	0.68	0.60	2.40	2.28	2.29	2.25
Thd	0.63	0.54	2.22	2.18	2.20	2.15
Cyd	0.50	0.49	2.22	2.15	2.60	2.12
Guo	0.37	0.07	1.72	1.65	1.70	1.63
Ado	0.13	0.01	1.86	1.77	1.85	1.74

AEA as a function of the simulation time

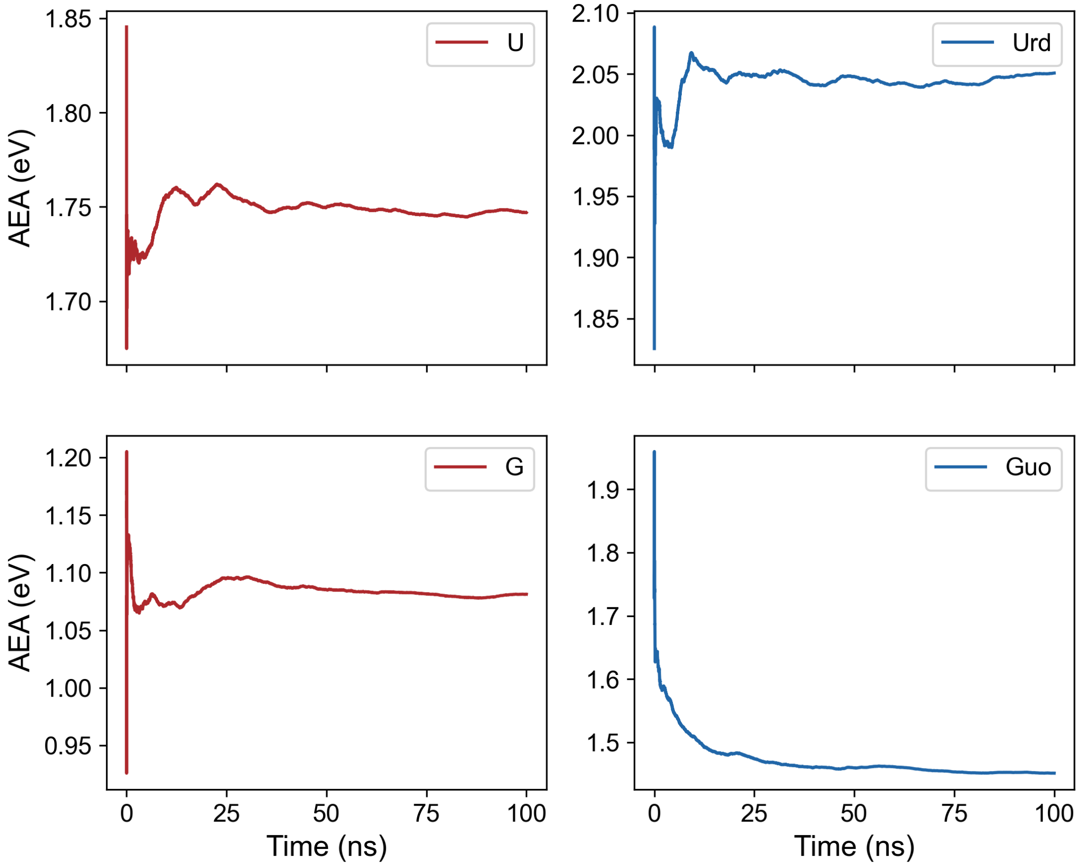

Figure S6: PMM-MD calculated AEA of uracil (U) and guanine (G) nucleobases and uridine (Urd) and guanosine (Guo) nucleosides in DMF solution as a function of the simulation time (frames) used for the calculation of this property. Similar convergence properties were found for the other compounds.

Analysis of the inhomogeneous perturbing field of the solvent from PMM-MD calculations

Table S4: Effects of the environment (water or DMF) on the mean electronic energies for the neutral ($\langle \mathcal{U}_{e,ox} \rangle_{ens} - \mathcal{U}^0_{e,ox} = \Delta^{ens}_{ox}$) and radical anion ($\langle \mathcal{U}_{e,red} \rangle_{ens} - \mathcal{U}^0_{e,red} = \Delta^{ens}_{red}$) states in eV. The estimated standard error is ± 0.04 eV.

		Neutral Ensemble		Anion Ensemble	
		Δ^{ox}_{ox}	Δ^{ox}_{red}	Δ^{red}_{ox}	Δ^{red}_{red}
**DMF**	U	-0.92	-0.56	-0.37	-4.11
	T	-0.88	-0.55	-0.37	-4.05
	C	-1.25	-0.81	-0.57	-4.34
	G	-1.53	-1.09	-0.89	-4.34
	A	-0.84	-0.60	-0.40	-4.01
	Urd	-0.69	-0.62	-0.48	-4.52
	Thd	-0.65	-0.58	-0.44	-4.41
	Cyd	-1.05	-1.04	-0.89	-5.07
	Guo	-1.17	-1.10	-0.98	-4.79
	Ado	-0.67	-0.87	-0.58	-4.64
**Water**	U	-1.44	-1.21	-1.80	-6.95
	T	-1.55	-1.25	-1.74	-6.64
	C	-1.70	-1.55	-2.14	-7.14
	G	-2.21	-1.98	-2.50	-7.23
	A	-1.38	-1.32	-2.15	-6.53
	Urd	-1.17	-1.28	-1.75	-7.12
	Thd	-1.19	-1.29	-1.71	-6.84
	Cyd	-1.57	-1.80	-2.16	-7.45
	Guo	-1.67	-1.76	-2.17	-7.19
	Ado	-1.09	-1.43	-1.79	-6.89

Reduction Potentials at the CCSD level of theory

Table S5: PMM-MD calculated reduction potentials of the nucleosides in DMF using CAM-B3LYP/6-311++G(2d,2p) and CCSD/aug-cc-pVDZ level of theory for the electronic structure calculations. The CAM-B3LYP/6-311++G(2d,2p) estimates are also reported here for comparison. Values are in V and reported against SHE. The estimated standard error on the calculated reduction potentials is ± 0.07 V.

Nucleoside	E (V)	
	CAM-B3LYP	CCSD
Urd	-2.19	-2.48
Thd	-2.31	-2.40
Cyd	-2.27	-2.38
Guo	-2.91	-2.88
Ado	-2.77	-[a]

[a]It was not possible to find the minimum of the adenine VB radical anion at CCSD/aug-cc-pVDZ level of theory.

References

(1) Aschi, M.; Spezia, R.; Di Nola, A.; Amadei, A. A first-principles method to model perturbed electronic wavefunctions: the effect of an external homogeneous electric field. *Chem. Phys. Lett.* **2001**, *344*, 374–380.

(2) Spezia, R.; Aschi, M.; Di Nola, A.; Amadei, A. Extension of the perturbed matrix method: application to a water molecule. *Chem. Phys. Lett.* **2002**, *365*, 450–456.

(3) Zanetti-Polzi, L.; Del Galdo, S.; Daidone, I.; D'Abramo, M.; Barone, V.; Aschi, M.; Amadei, A. Extending the perturbed matrix method beyond the dipolar approximation: comparison of different levels of theory. *Phys. Chem. Chem. Phys.* **2018**, *20*, 24369–24378.

(4) Isse, A. A.; Gennaro, A. Absolute potential of the standard hydrogen electrode and the problem of interconversion of potentials in different solvents. *J. Phys. Chem. B* **2010**, *114*, 7894–7899.

(5) Groenhof, G. *Biomolecular Simulations*; Springer, 2013.

(6) Singh, U. C.; Kollman, P. A. An approach to computing electrostatic charges for molecules. *J. Comput. Chem.* **1984**, *5*, 129–145.

(7) Besler, B. H.; Merz Jr, K. M.; Kollman, P. A. Atomic charges derived from semiempirical methods. *J. Comput. Chem.* **1990**, *11*, 431–439.
